# Extracellular vesicles in pancreatic cancer progression and therapies

**DOI:** 10.1038/s41419-021-04258-7

**Published:** 2021-10-20

**Authors:** Chao-Hui Chang, Siim Pauklin

**Affiliations:** Botnar Research Centre, Nuffield Department of Orthopaedics, Rheumatology and Musculoskeletal Sciences, University of Oxford, Windmill Road, OX3 7LD Oxford, UK

**Keywords:** Cancer microenvironment, Cancer stem cells

## Abstract

Pancreatic cancer (PC) is one of the leading causes of cancer-related death worldwide due to delayed diagnosis and limited treatments. More than 90% of all pancreatic cancers are pancreatic ductal adenocarcinoma (PDAC). Extensive communication between tumour cells and other cell types in the tumour microenvironment have been identified which regulate cancer hallmarks during pancreatic tumorigenesis via secretory factors and extracellular vesicles (EVs). The EV-capsuled factors not only facilitate tumour growth locally, but also enter circulation and reach distant organs to construct a pre-metastatic niche. In this review, we delineate the key factors in pancreatic ductal adenocarcinoma derived EVs that mediate different tumour processes. Also, we highlight the factors that are related to the crosstalk with cancer stem cells/cancer-initiating cells (CSC/CIC), the subpopulation of cancer cells that can efficiently metastasize and resist currently used chemotherapies. Lastly, we discuss the potential of EV-capsuled factors in early diagnosis and antitumour therapeutic strategies.

## Facts

- PDAC-derived exosomes show heterogeneity in their cargos.
- PDAC-derived exosomes distinctly regulate angiogenesis, cancer-associated thrombosis, immune evasion, stromal reprogramming, chemoresistance, metastasis and tumour growth during PDAC tumorigenesis.
- CSC/CIC-derived exosomes transmit signals for efficient metastasis and for creating a pre-metastatic niche in the distant organ.
- Around 10–20% of identified exosomal proteins are transcription factors and nucleotide-binding factors.

## Open questions

- How do EVs mediate the pivotal crosstalk in the microenvironment to regulate the main hallmarks of tumorigenesis?
- How do EVs modulate distant organs that will home metastases?
- Can EVs mediate intercellular epigenetic regulation between cells and different cell types through transmitting transcription factors and nucleotide-binding factors to recipient cells?
- Which EV cargo proteins or nucleic acids can be used as reliable markers for early detection and prognosis of pancreatic cancers from blood or other sources?
- Will a combination of current biomarkers and the exosomal proteins provide better sensitivity and specificity of early cancer detection?
- Can exosome-based therapies improve therapeutic outcomes in pancreatic cancer?

## Pancreatic ductal adenocarcinoma (PDAC) and extracellular vesicles (EVs)

Pancreatic ductal adenocarcinoma (PDAC), the most common type of pancreatic cancer, remains one of the deadliest cancers in the world. This is due to its poor diagnosis at early stages, high risk of metastasis, and limited response to treatments, including surgery and chemotherapy [1]. Surgical resection is the potential curative therapy and chemotherapy drugs serve as the first-line treatment for patients. Nevertheless, surgical resection is usually followed by cancer recurrence, and chemotherapy resistance commonly appears in patients with rapid tumour progression [1, 2]. It is reported that the 5-year survival rate for PDAC patients made up <10% and the median survival time is 5–6 months [1].

**Fig. 1:**
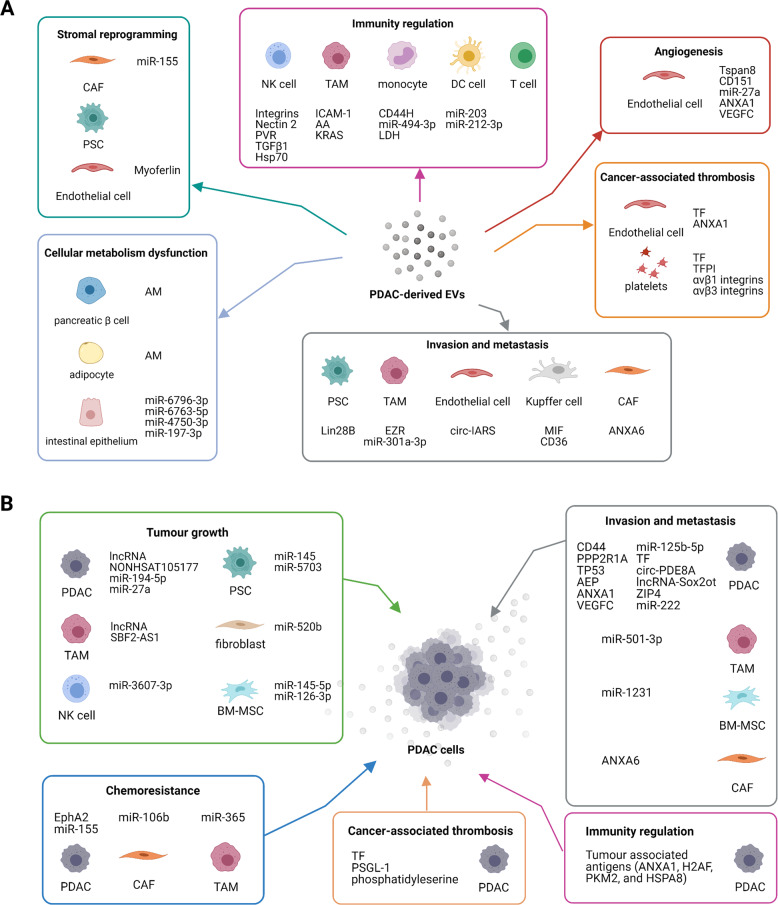
The EV-mediated communication between PDAC and tumour microenvironment. **A** PDAC-derived EVs contain various cargos that selectively modulate the surrounding cells types. **B** Different cell types exist in PDAC microenvironment and produce EVs with various contents that regulate PDAC progression. PDAC pancreatic ductal adenocarcinoma, PSC pancreatic stellate cell, TAM tumour-associated macrophage, CAF cancer-associated fibroblast, BM-MSC bone marrow-derived mesenchymal stem cell, NK natural killer cell, DC dendritic cell.

Tumour progression is driven by the communication between tumour cells and the surrounding cells in the microenvironment, as well as the distant organs. It can be mediated by direct cell–cell contacts, secretory proteins, and extracellular vesicle (EVs). EV is defined as a lipid bilayer particle that is naturally secreted by cells into extracellular microenvironment without replication capability. EVs display in a wide range of size, biogenesis, biochemical composition and biological functions, including cell–cell communication, immune response modulation, and disease progression [3]. Based on the particle sizes and origins, EVs are generally divided into three groups: exosomes (diameter from 30 to 150 nm), microvesicles (microparticles or ectosome, diameter from 50 to 1000 nm), and apoptotic bodies (diameter from 500- over 1000 nm) [4]. Though EV release is a normal process, an increasing rate in their release and change in cargos such as DNA, RNA, miRNA and proteins, can favour cancer development. Over the course of tumorigenesis, exosomes are generated from the tumour cells as well as from the surrounding stromal cells, such as cancer-associated fibroblasts (CAFs), tumour-associated macrophages (TAMs) and bone marrow mesenchymal stem cell (BMSCs). These EVs deliver cargos to different recipients which facilitates tumour progression and tumour niche construction, through regulating angiogenesis, cellular metabolism, metastasis, cell survival, immune regulation and therapeutic resistance (Fig. 1).

We summarise the current understanding of the role of EVs in pancreatic tumour progression and discuss the potential strategy of using EVs in cancer treatment (Table 1).

**Table 1:** Summary of EV relevant literature search from PubMed till 24 June 2021.

	Number of articles
Queries in PubMed	
Exosome(s) and pancreatic cancer	423
Extracellular vesicles and pancreatic cancer	349
Extracellular vesicle and pancreatic cancer	359
Excluding articles	
Duplicate searching from the three queries	567
Non-relevant articles	100
Non-English articles	5
Results	459
Reviews/Comments/Editorials etc.	158
EV secretion/fusion mechanism	20
Drug-loaded EV as a therapy	18
miRNA, transcriptomic or proteomic profiling	47
Biomarkers or EVs detection methods for Clinics	114
EV original research articles	102

The search was conducted using combined synonyms for ‘extracellular vesicle(s)’ or ‘exosome(s)’ and ‘pancreatic cancer’ in titles and abstracts to identify original articles published until 24 June 2021.

## PDAC-derived EVs

### PDAC-derived EVs regulate angiogenesis

Communication between tumour cells and endothelial cells leads to new blood vessel formation that supports tumour growth. PDAC-derived EVs induce HUVEC cell proliferation and mobility, as well as influence their proangiogenic factor secretion [5–7]. Tetraspanins are a family of proteins with four transmembrane domains that are abundant in membranes of organelles and in exosomes. Two tetraspanins, CD151 and tetraspanin 8 (also known as CO-029 and TM4SF3 in human, and D6.1A in rats), are upregulated in PDAC and support tumour progression. Overexpression of D6.1A in PDAC promotes angiogenesis in vivo through transmitting PDAC-derived exosomal D6.1A to endothelial cells and consequently induces the expression of angiogenic factors such as VEGFR and CD31 [8]. CD151 selectively affects angiogenesis [9, 10]. Assembly defects during skin wound healing and tubular formation are observed in *cd151* knockout and *tspan8* knockout mice, and even more severe effects in the double knockout mice [9]. Interestingly these impaired phenomena in knockout mice can be mitigated by Tspan8/CD151-competent serum exosomes, through stimulating GPCR and RTK-dependent angiogenesis which ultimately contribute to tumour progression in vivo [9]. This suggests a potential application of joint Tspan8 and CD151 blockage in cancer therapeutics.

PDAC cell-derived exosomal miR-27a promotes angiogenesis through upregulating the expression of angiogenesis factors VEGF and VEGFR, and migration-related factors MMP2 and MMP9 in tumour cells [11]. Another PDAC-derived exosomal protein, ANXA1, positively regulates endothelial tubular formation [12] and fibroblast and endothelial cell mobility [13]. Similarly, PK-45H cell-derived exosomes promote in vitro HUVEC tube formation via phosphorylation of Akt and Erk signalling pathway and dynamin-dependent endocytosis [14]. Tumour-derived exosomes also increase permeability of endothelial monolayer cells via transferring circ-IARS into recipient cells and activating their miR122/RhoA signalling, thereby leading to upregulation of F-actin and downregulation of ZO-1 and facilitating tumour metastasis [15]. Depletion of myoferlin influences exosome production in PDAC and alleviates exosome entry into HUVEC, decreasing proliferation and migration of endothelial cells [16].

A recent study showed that DUSP2/VEGF-C axis in PDAC mediated lymphangiogenesis and tumour invasion [17]. Inhibition of DUSP2 increased VEGF-C expression in PDAC-derived exosomes [17]. These VEGF-C-containing exosomes promoted proliferation of lymph endothelial cells and lymphovascular invasion in the orthotopic PDAC mouse model [17].

### PDAC-derived EVs deliver tissue factor (TF) promoting cancer-associated thrombosis

Cancer-associated thrombosis is the most common complication in patients that causes high mortality. It shows a higher association with certain cancer types, such as PDAC, lung cancer, brain cancer, kidney cancer, lymphoma and ovarian cancer, while the reasons remain unclear. Tissue factor (TF) is an initiator of the coagulation cascade and is essential for haemostasis. Under normal condition, TF is not expressed by the cells that directly contact with blood, except a low level in CD14^+^ monocytes, to prevent inappropriate activation of coagulation in the absence of vascular injury. In cancers, TF is expressed by tumour cells as an extracellular secretory factor or EV cargo. PDAC-derived EVs carrying TF can trigger venous thrombosis in mice [18] and foster prothrombotic activity in assistance of phospholipids [19, 20]. This prothrombotic activity can occur in platelet-dependent manner [21–23], and cooperate with neutrophils that release neutrophil extracellular trap (NET) [24]. NETs serve as a scaffold for platelet adhesion and subsequently initiate coagulation. On the other hand, Stark and colleagues showed that initiation of thrombosis was a platelet- and leucocyte-independent mechanism which required phosphatidylethanolamine-dependent TF^+^EVs from host PDAC cells [19].

### PDAC-derived exosomes regulate pancreatic functions

High prevalence of PDAC-associated diabetes is a hallmark of PDAC progression. Many patients report dramatic weight loss and diabetes indicating that tumour cells disrupt normal pancreatic metabolism. The PDAC-derived exosomal adrenomedullin (AM) induces p38 and ERK1/2 MAPK-mediated lipidosis in adipocytes [25], and inhibits insulin secretion by β-cells [26]. Also, ER stress markers, ROS and NO are upregulated in β-cells upon PDAC-derived exosomal AM treatment [26]. Mechanically, AM interacts with its receptor, ADMRs, to initiate the dysfunction of the recipient cells, and blockage of ARMRs abrogates these effects. An independent study showed that lipidosis and glucose intake inhibition caused by PDAC-derived exosomes is PI3K/Akt/FoxO1 signalling dependent, which eventually develops insulin resistance in skeleton muscle cells [27]. PDAC-derived exosomes suppress the synthesis of glucose-dependent insulinotropic peptide (GIP) and glucagon-like peptide-1 (GLP-1) by downregulating the proprotein convertase subtilisin/kexin type 1/3 (PCSK1/3) [28]. Insulin resistance in skeleton muscle cells together with impaired GIP and GLP-1 are involved in pathogenesis of type 2 diabetes mellitus. Further investigations in the relationship between PDAC and systemic metabolism will help to discover potential targets for treating metabolic disorders and improving the diagnosis/treatment in PDAC.

### PDAC-derived exosomes in immune regulation

An imbalance of immune cells in PDAC microenvironment leads to an immunosuppressive and protumorigenic phenotype. These include immunosuppressive T_reg_ lymphocytes, M2 polarized TAM and myeloid-derived suppressor cells (MDSCs) that prevail over immune effector CD8^+^ T cells, dendritic cells (DCs) and M1 polarized TAM in the tumour microenvironment, blood and lymphoid organs. PDAC-associated immune cell alterations concur with immune escaping, hence favouring tumour progression. Lymphocyte lineage alterations with T_reg_ cell accumulation appear to occur early, while myeloid lineage with MDSCs accumulation occur later during PDAC development. Such a T_reg_ cell expansion is mediated by PDAC-derived EVs through upregulating the expression of FOXO transcription factors and nuclear translocation in FOXP3^+^ T_reg_ cells [29].

PDAC-derived exosomes cause an imbalance of immune cells via expanding monocytic myeloid-derived suppressor cells (mMDSCs; CD11b^+^CD14^+^HLA-DR−), while reducing DCs (CD11b^+^CD14^−^HLA-DR^+^) and granulocytic MDSCs (gMDSCs; CD11b^+^CD14^−^HLA-DR^−^) [30]. These exosomes increase intracellular calcium trafficking in a SMAD4-dependent manner in mMDSCs and induce aerobic glycolysis in a SMAD4-independent manner in PBMCs [30]. An increase in CD14^+^HLA-DR^lo/neg^ monocyte population was confirmed in PDAC patients by tumour-derived exosomes that involves an alteration in STAT3 signalling, induction of arginase expression and ROS generation [31]. Also, tumour-derived exosomes can regulate monocytes via CD44H, a transmembrane protein on monocytes, that initiates phagocytosis and chemokine production, namely CCL5 and CCL4, via STAT3-mediated signalling [32].

Using rat PDAC-derived exosomes, Zöller et al. showed that these exosomes enter all leukocyte subpopulations and strengthen cytotoxic T cell and NK cell activity [33]. In line with these findings, PDAC exosomes enriched in heat shock protein 70 (Hsp70) stimulate NK cell migration and trigger their cytotoxicity against tumour cells via releasing granzyme B [34]. By contrast, PDAC-derived EVs have also been reported to attenuate NK cell cytotoxicity by downregulating the expression of NKG2D, CD107a, TNFα and INFϒ, as well as impairing their glucose uptake [35]. PDAC patient serum-derived EVs are TGFβ1 enriched and able to induce SMAD2/3-dependent signalling in NK cells, suggesting a potential distant immunosuppression by PDAC-derived exosomes [35]. Similarly, saliva exosomes from pancreatic tumour-bearing mice suppress natural killer (NK) cell cytotoxic capability against tumour cells [36]. PDAC-derived exosomes activate p38 MAPK in T lymphocytes leading to ER stress-mediated apoptosis and ultimately immunosuppression [37].

PDAC-derived exosomal miR-203 is transferable to DCs and decreases their toll-like receptor 4 (TLR4) expression, TNFα and IL-12 secretion [38]. A further report showed a reduction of regulatory factor X-associated protein (RFXAP) in DCs by PDAC-secreted exosomal miR-212-3p, resulting in decreased MHC II expression and induced immune tolerance [39].

PDAC-derived exosomes deliver arachidonic acid (AA) to non-polarised THP-1 macrophages (M0) to induce a M2-like phenotype (CD14^hi^CD163^hi^CD206^hi^) and increase a panel of pro-tumoral factors, including VEGF, MCP-1, IL-6, IL-1β, MMP-9 and TNFα [40]. KRAS is another extracellular molecule transferred by PDAC-derived exosomes to macrophages to cause M2-like phenotype switch via STAT3 dependent fatty acid oxidation [41].

B-cell-associated immune response is reported in many cancers including PDAC. An accumulation of B-cell subpopulation has been observed during early pancreatic neoplasia [42] and in HIF-1α depleted PDAC model [43], with a positive correlation to pancreatic tumorigenesis. Bruton tyrosine kinase, a key B-cell and macrophage kinase [44], and PD-1/PD-L1 pathway [45], suppress T-cell-dependent antitumour immune responses in PDAC, indicating potential mechanisms of B cells in favour of tumour progression. Although these murine studies have indicated a pro-tumour role for B cells, the clinical data are controversial. For instance, higher B-cell infiltration within PDAC tumours related to shorter survival time is reported in some studies [45, 46], whereas others state differently [47–49]. The detailed roles of B cells in PDAC need further investigations. B-cell-associated autoimmune response is evidenced by the production of autoantibodies against tumour-associated antigens (TAAs). Capello et al. have described a panel of TAAs, such as LGALSBP3, PKM2, HSPA8, KRT17, KRT16, ACTB, H2AF, KRT5 and JUP, which was enriched on the surface of PDAC plasma-derived exosomes vs healthy donors [50]. Moreover, the exosomal antigens induce and bind to autoantibodies in PDAC patients, indicating PDAC-derived exosomes inhibit complement-dependent cytotoxicity towards tumour cells [50]. This suggests a decoy-like function of PDAC-derived exosomes by binding to circulating autoantibodies in patient serum and attenuating B-cell-associated antitumour responses, hence favouring an immune escape.

### PDAC-derived exosomes deliver chemoresistance

Chemoresistance happens during long-term exposure to chemotherapeutic drugs. As it usually occurs rapidly and comprehensively, an efficient mechanism such as exosomes has been proposed to spread the signals in tumour cells. In this case, exosomal cargos from chemoresistant cells can regulate gene expression in the chemosensitive recipient cells to support their survival or anti-apoptotic capacity against chemotherapy. Gemcitabine is a primary cancer drug that can change tumour cell contents including their derived exosomes. miR-155 is a well-studied onco-miRNA that has been found to be overexpressed in most PDAC cases and to correlate with a poor survival of PDAC patients. Upregulation of miR-155 in PDAC cells and exosomes by gemcitabine can be delivered to other PDAC cells to protect against gemcitabine-induced cell death in vitro and in NOD/SCID mice [51, 52]. Deoxycytidine kinase (DCK) is one of miR-155 targets. Upon chemotherapy, DCK converts gemcitabine into an active form which incorporates into DNA and inhibits DNA synthesis, consequently leading to tumour cell apoptosis. Downregulation of DCK by miR-155 halts this process and supports PDAC cell survival [51, 52]. TP53INP1, a pro-apoptotic stress-induced p53 target gene, was also decreased in PDAC cells upon the uptake of exosomal miR-155 [51, 52]. Hence, upregulation of miR-155 represents a protection mechanism by tumours against chemotherapy and an indicator of poor survival. EphA2 protein is another example. Gemcitabine-resistant PDAC cells secrete EphA2 enriched exosomes that can be transmitted into gemcitabine-sensitive PDAC cells to support their survival [53]. Both miR-210 and exosomal miR-210 expression is upregulated in PDAC by gemcitabine and the latter is also transferrable from a chemoresistant CD133^+^CD44^+^ PDAC cell population to a chemosensitive PDAC cell population [54]. Exosomal miR-210 promotes tumour growth in nude mice via activating mTOR signalling [54].

### PDAC-derived exosomes promote invasion and metastasis

The exosomes collected from advanced PDAC patient serum enhance PDAC cell migration and proliferation in vitro, increase CD44 and PPP2R1A expression and decrease TP53 expression in recipient PDAC cells [55]. Asparaginyl endopeptidase (AEP), highly abundant in PDAC patient sera, shows positive effects on PDAC cell invasion [56]. Such an invasive ability is transferrable between tumour cells. In vitro studies using paired PDAC cell lines show that metastatic ability is able to be transferred to their less motile counterparts through exosomes cargos, such as miR-125b-5p [57], ZIP4 [58], circular-RNA-PDE8A [59], miR-222 [60] or lncRNA-Sox2ot [61–63].

#### Long-distant metastasis niche

Liver metastases are reported in up to 80% of PDAC patients and lung metastases occur in 45% of PDAC patients [64]. Pre-treating the mouse with PDAC-derived exosomes has been shown to enhance tumour growth and tumour metastasis [63]. Costa-Silva and colleagues showed that pre-injection of PDAC-derived exosomes in mice ‘educated’ Kupffer cells to enhance the metastatic burden in liver [65]. These PDAC-derived exosomes fused with Kupffer cells and changed their ECM components by increasing fibronectin and decreasing vitronectin and tenascin C [65]. It also induced liver fibrosis signalling molecules, such as CTGF and TGFβ, and recruited bone marrow-derived F4/80 macrophages to the liver pre-metastatic niche [65]. In their study, also migration inhibitory factor (MIF) was identified in exosomes for such liver pre-metastatic niche construction [65]. Tumour-derived exosomes deliver cargos into macrophages via CD36, a lipid receptor, to create a pre-metastatic niche in mouse model [66].

Emmanouilidi et al. revealed that oncogenic exosomes contain factors known to regulate the pre-metastatic niche (S100A4, F3, ITGβ5, ANXA1), invasion (PODXL, ITGA3) and metastasis (LAMP1, ST14) [67]. Protein Kinase D1 (PRKD1) [68] and p53 [69] are reported in PDAC-derived exosomes for lung metastases. Reduced expression of PRKD1 in PDAC increases their exosome secretion and promotes lung metastasis in mice [68]. Mutant p53-expressing exosomes from tumour cells can transfer invasive/migratory ability to other tumour cells as well as regulate lung ECM to facilitate their invasion [69]. Hypoxia regulates tumour mobility through exosomal cargos. Wang et al. showed that hypoxic exosomes derived from PDAC activate macrophages to the M2 polarity, which in turn promoted tumour metastasis to lung [70]. The exosomal miR-301a is upregulated by hypoxia-inducible factors (HIF) and transmitted into macrophages for their activation via stimulating PI3K signalling [70]. Earlier, Zöller’s group revealed both CD44v6 and soluble matrix were necessary for lung pre-metastatic niche formation [71]. Similarly, exosomal CD44v6/complement C1q binding protein (C1QBP) also activates hepatic satellite cell for liver metastasis and liver fibrosis in mice [72].

### PDAC-derived exosomes in tumour growth and progression

Pancreatic cancer cell-derived exosomes show potential to support malignant cell transformation compared to normal pancreatic cell-derived exosomes [73]. Verince’s group reported that PDAC-derived EVs induced mitochondria-dependent apoptotic pathway in tumour cells through PTEN and GSK-3β activation [74], and downregulation of Hes-1 expression, a Notch-1 signalling pathway mediator [75]. In these studies, the decrease in Hes-1 expression by presenilin blockage resulted in PTEN and GSK-3β activation [74], whereas inhibiting either PTEN or GSK-3β activation increases Hes-1 expression which counteracts their effects on inhibiting tumour cell proliferation [75]. This suggests a complexity in signalling crosstalk induced by EVs, whereas the effects may depend on where the vesicles anchor and trigger the intracellular signalling in recipient cells. Another study has identified a group of long non-coding RNAs (lncRNAs), including NONHSAT105177, capable of mediating drug inhibition on PDAC proliferation via cancer-derived exosomes [76]. Overexpressing NONHSAT105177 significantly downregulates cholesterol pathway genes, and inhibits PDAC cell proliferation, migration, and the epithelial–mesenchymal transition (EMT) both in vitro and in vivo [76]. Exosomal lncRNA 01133 (LINC01133) [77], has-circ-0000069 [78] and Survivin [79] have also been recently reported in tumour progression and may impact treatment.

## EV-mediated crosstalk between tumour cells and stroma

### Between tumour cells and pancreatic stellate cells (PSCs)

PSCs play a pivotal role in pancreatic fibrogenesis to develop a conditioned microenvironment for tumour growth. PDAC-derived exosomes stimulate proliferation, migration and profibrogenic activity of PSCs, potentially via transforming growth factor β1 (TGFβ) and tumour necrosis factor (TNF) that mediate ERK and AKT activation [80]. miR-1246 and miR-1290 are abundantly expressed in PDAC-derived exosomes and induce α-smooth muscle actin (ACTA2) and fibrosis-related genes in PSCs to support tumour growth [81]. PDAC-derived exosomal Lin28B protein chemoattracts PSC migration, which in turn supports tumour progression in mice [82]. Co-incubation of PSC-derived exosomes with PDAC cells induces their chemokine gene expression, such as CXCL1, CXCL2 and CCL20, and stimulates PDAC proliferation and migration [83]. Also, hypoxia regulates PSC-derived exosomal miRNAs in favour of tumour progression and invasion [84]. By contrast, a stroma specific miR-145 can be delivered in exosomes to tumour cells that induces apoptosis and suppresses tumour cell growth [85]. Furthermore, PSC-derived exosomal miR-5703 inhibits PDAC proliferation via targeting *CMTM4* expression [86], which in turn suppressed PI3K/Akt pathway through downregulating PAK4 [86].

### Between tumour cells and CAFs

PDAC-derived microvesicles containing miR-155 promote conversion of normal fibroblasts to CAFs by downregulating TP53INP1 expression and upregulating the expression of a-SMA, fibroblast activation protein (FAP), and fibroblast growth factor 2 [81, 87]. These profibrogenic activities eventually facilitate tumour spreading. Furthermore, CAFs are innately drug resistant and able to transfer such ability to their neighbouring tumour cells via exosomes [88, 89]. CAF-derived exosomes support PDAC cell growth upon gemcitabine treatment in vitro [88]. miR-106b carried in CAF-derived exosomes blocks apoptosis through targeting TP53INP1 in PDAC cells, though the detailed mechanism is still unclear [89]. Metabolically, CAF-derived exosomes inhibit mitochondrial oxidative phosphorylation in tumour cells, thereby increasing glycolysis and glutamine-dependent reductive carboxylation to support tumour growth [90]. In hypoxic and lipid starvation condition, like a tumour microenvironment, co-culture of CAFs with macrophages facilitates ANXA6/LRP/TSP1 complex formation in CAFs [91]. This complex is subsequently delivered in exosomes to PDAC cells enhancing their migration ability and tumour progression [91]. Manipulating ANXA6 expression in CAFs in a co-culture with PDAC cells correlates with liver metastasis in xenografts, suggesting a potential of ANXA6^+^ EVs as a biomarker for PDAC aggressiveness [91]. In addition, a number of oncogenic miRNAs (miR-10a-5p, miR-92a-3p, miR-181a-5p, miR-191–5p, and miR-92b-3p) have been identified in CAF-derived EVs that support tumour migration [92]. In contrast, the exosomal miRNA-520b derived from normal fibroblasts, inhibits PDAC cell proliferation, invasion, migration and stimulates apoptosis, suggesting its therapeutic potential in PDAC treatment [93].

### Between tumour cells and TAMs

A higher expression level of exosomal Ezrin protein is observed in PDAC patients and pancreatic ductal cell lines [94]. Enriched exosomal Ezrin protein can regulate M2 macrophage polarity which consequently leads to tumour metastasis [94]. Interestingly, exosome containing Ezrin promotes M2 marker expression and cytokine production while direct incubation of Ezrin with macrophages showed no statistical significance. Injection of patient-derived exosomes that expressed Ezrin before and post tumour injection into mouse models facilitated tumour metastasis to liver. This suggests that specific exosomal proteins derived from tumour cells could regulate stromal cells in the tumour microenvironment to support tumour spreading.

TAMs-derived exosomes support PDAC cell survival in response to gemcitabine via interfering with gemcitabine activation [95]. Chitinase 3-like-1 (CHI3L1) and fibronectin (FN1) are two abundant proteins in macrophage-derived EVs that contribute to gemcitabine resistance [96]. In addition, the uptake of TAM-derived exosomal miR-365 by PDAC cells increases the expression of cytidine deaminase (CDA), an enzyme that deaminates gemcitabine, and excretes gemcitabine out of the cells [95]. miR-365 has been previously reported to directly target SHC1 and BAX to block apoptosis in PDAC upon gemcitabine treatment, indicating that the miR-365/SHC1/BAX axis modulates survival of PDAC cells [97]. Exosomal miR-365 from M2 macrophages not only regulates chemoresistance but it also promotes PDAC development through suppressing B-cell translocation gene 2 (BTG2) expression and activating FAK/AKT pathway in tumour cells [98]. Together, M2-derived exosomal miR-365 exerts distinct functions in tumour cells to support cancer progression.

M2 macrophages secrete exosomes that regulate gene expression which promote PDAC migration, invasion and angiogenesis [99]. miR-501-3p supports tumour formation and liver metastasis in nude mice via downregulating TGFBR3 in recipient PDAC cells while upregulating TGFBR1 and TGFBR2 [99]. Blocking miR-501-3p reduces tubular formation, tumour formation and metastasis to liver, as well as downregulates stemness-related genes, such as CD133, OCT4 and NANOG [99]. Constrained lncRNA-SBF2-AS1 in M2 macrophage-derived exosomes increases miR-122-5p expression to reduce XIAP expression in tumour cells leading to tumour suppression [100].

### Other immune cell-derived exosomes suppress PDAC progression

There is also crosstalk between PDAC cells and other stromal cells. miR-3607-3p is enriched in the NK cell-derived EVs while a low level of miR-3607-3p correlates with poor diagnosis in PDAC patients [101]. Exosomal miR-3607-3p from NK cells targets IL-26, and thereby inhibit proliferation, migration and invasion of PDAC cells. miR-1231 derived from Bone marrow mesenchymal stem cells (BM-MSCs) negatively correlates with PDAC tumour progression and its downregulation shortens cell cycle progression and enhances PDAC cell migration/invasion [102]. Ding et al. applied exosomes from human umbilical cord mesenchymal stromal cells (hucMSCs) to deliver exogenous miR-145-5p to pancreatic cells and inhibited cell proliferation and invasion, while inducing cell cycle arrest and apoptosis in vitro and in vivo [103]. Exosomal miR-126-3p derived from primary BM-MSC inhibited PDAC cell proliferation, migration, invasion, and promoted their apoptosis [104]. Exosomal miR-143 [105] and miR-124 [106] from BM-MSCs have shown similar suppression of PDAC progression.

## Exosomes and cancer stem cell/cancer-initiating cell (CSC/CIC)

A cancer stem cell (CSC), alternatively cancer-initiating cell (CIC) or tumorigenic cell, is defined as a cell within a tumour that possesses the capacity to self-renew and divide asymmetrically to cause the heterogeneous lineages of cancer cells that constitute the bulk of the tumour [107]. CSCs represent a side-population in the tumour which exerts stem cell-like characteristics such as entering a quiescent state, slow cell cycling, expressing stemness markers and stemness transcription factors. These together make CSCs responsible for the maintenance and proliferation of the tumour, enhance metastatic characteristics and resistance to chemo- and radiotherapy, which ultimately lead to cancer relapse. Though the specificity remains controversial, several CSC markers have been reported in PDACs, including CD133, CD24, CD44, CXCR4, EpCAM, ABCG2, c-Met, ALDH-1 and Nestin [108].

**Fig. 2:**
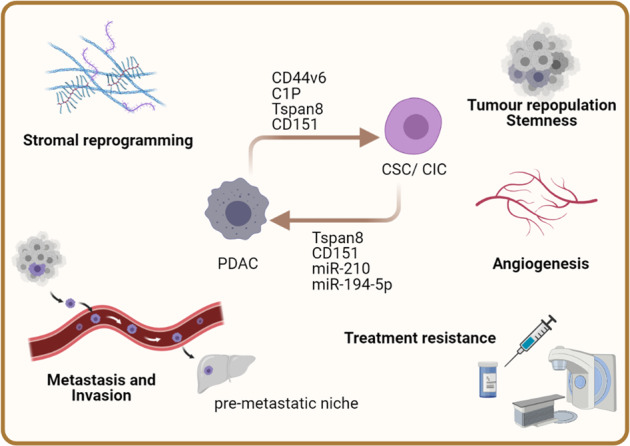
The EV-mediated communication between PDAC and cancer stem cell/cancer-initiating cell (CSC/CIC). PDAC cell-derived EVs contain cargos such as CD44v6, C1P, Tspan8 and CD151, while CSC/CIC-derived EVs carry factors including Tspan8, CD151, miR210 and miR-195-5p that regulate biological functions in the counterparts. This includes upregulation of stemness and survival genes in the recipient cells that provide resistance to radiation and chemotherapies, stimulate protein secretion for stromal reprogramming and angiogenesis, and the switching to metastatic phenotype for metastasizing to distant sites.

Increasing evidence suggests that exosomes derived from CSCs promote survival, EMT and motility in tumour cells, and angiogenesis in endothelial cells (Fig. 2). These ultimately promote tumour progression. Wang et al. reported Tspan8, CD44v6 and α6β4 as biomarkers for PDAC CIC [109]. As mentioned previously, Tspan8/CD151 exosomes contribute to tumour progression by stimulating angiogenesis in vivo [10]. In addition, PDAC-derived exosomal CD151 and Tspan8 induce matrix remodelling in recipient cells via exosomal tetraspanin–integrin and tetraspanin–protease associations, which ultimately promote the metastatic phenotype [110]. Similarly, CD151 and Tspan8 defects cause reduction of exosome uptake and therefore weaken their biological functions in recipients.

The same group also reported that CD44 variant isoform v6 (CD44v6), a known CIC marker and pro-metastatic molecule, contributes to PDAC migration and invasion [111]. Genetically knocking down CD44v6 concomitantly decreased the expression of CD151 and Tspan8, whereas loss of CD44v6 and Tspan8 lead to decreased invasiveness and lower protease expression. CD44v6 mediates exosome uptake by recipients while its deficiency can be reversed by CD44v6 wild-type exosomes and even enhanced by the exosomes derived from spheroids or holoclonal cells (a colony-forming stem cell that has a higher growth potential and does not contain differentiated cells). Furthermore, the PDAC CIC-derived exosomes promote various stem cell-like features, including apoptosis-resistance, in vivo tumour growth and progression, and inducing EMT gene expressions in CD44v6^kd^/Tspan8^kd^ non-CIC cells via initiating receptor tyrosine kinase (RTK) signalling [112]. Recently, Jiang and colleagues reported that exosomal miR-194-5p derived from dying tumour cells support ALDH1A1^+^ cell repopulation upon radiotherapy [113]. The exosomal miR-194-5p supports ALDH1A1^+^ cell survival via downregulating E2F3 expression and inducing G1/S arrest. It also triggers DNA damage response for DNA repair in irradiated ALDH1A1^+^ cells and facilitates their survival. In addition, treating with exosomal miR-194-5p recovers the expression of HMGA2 in irradiated ALDH1A1^+^ cells which promotes cell stemness and cancer progression.

An independent study showed that highly invasive PDAC cells secrete lncRNA-Sox2ot in exosomes which promote recipient PDAC epithelial–mesenchymal transition (EMT) and stem cell-like properties via regulating Sox2 expression [61]. PDAC cells secrete ceramide-1-phosphate (C1P)-containing exosomes that selectively stimulate CIC migration and adhesion on fibronectin [114]. On the other hand, exosomal miR-21-5p derived from M2 macrophage stimulates CSC/CIC differentiation through targeting KLF3 [115].

CSC-derived exosomes are believed to contribute to the communication in tumour microenvironment as well as long distant communication to other organs to promote tumour progression. Given the CSC biological characteristics, the CSC-derived exosomes can support tumour mass through delivering self-renewal capacity to one another and educating the recipients to regenerate multiple malignant cell types, as well as providing a niche for tumour growth. Moreover, CSCs can also transfer their material via exosome secretion to promote chemoresistance in other cancer cell subtypes [63].

## Exosomes and epigenetic regulation

Many studies show that exosomes regulate gene expression in receipt cells via delivering miRNAs and lncRNAs, whereas only a few have so far discussed a possible role of transcriptional regulators as exosomal cargo. PDAC related exosomal proteomic studies indicate that a minor proportion of identified proteins functionally associate with transcriptional regulation or translation. Between 10 and 20% of total identified exosomal proteins are usually residing in the cell nucleus, ranging in function from transcription or translation activity and nucleotide/DNA/RNA binding [116–118]. TP53, for instance, a widely known transcription regulator that is commonly mutated in late stage of PDAC, is present in plasma-derived exosomes from PDAC patients [119]. This suggests that the exosomes may be able to influence recipients’ behaviours not only with RNA-type cargo, but with proteins that directly regulate gene expression. In line with this hypothesis, a previous review indicates a similar proportion of identified proteins in human brain tumour or medulloblastoma cell line-derived exosomes has some role in transcriptional and/or translational activities [120]. Whether exosomes transport transcription factors or epigenetic regulators that could directly impact chromatin architecture and gene expression in recipients is still an open question. Since this has been suggested in other disease contexts it will be interesting to discover if such mechanisms are relevant for pancreatic cancer.

## Exosomes and early detection of PDAC

One of the main factors that causes the high mortality rate in PDAC is the lack of early detection markers. CA19-9 antigen, found about 35 years ago, is the most common indicator that is currently used for PDAC monitoring [121]. CA19-9 is regularly expressed on cells of the pancreatic-biliary system with low concentrations in healthy serum and elevated levels in cancer patients, particularly in PDAC. Although being routinely used in clinic, serum CA19-9 as a biomarker has its limitations, for example, the concentration can be interfered by other diseases, such as cholangitis, pancreatitis or obstructive jaundice, hepatic, and pancreatic cysts. Hence, other biomarkers are needed whether in combination with serum CA19-9 or separately to provide better diagnosis. A number of exosomal cargos including proteins, DNA, miRNAs, lncRNA, as well as exosomal membrane lipids, for instance, a well-known phosphatidylserine (PS) highly expressed on tumour cells and present in tumour-derived exosomes, have been proposed as potential biomarkers for PDAC [122].

Glypican-1 is upregulated in PDAC and breast cancer. Its elevated expression in circulating exosomes of pancreatic patients suggests its suitability as a biomarker for detection of early PDAC. Higher level of secretory and exosomal glypican-1 from serum is correlating with poor prognosis and tumour burden of PDAC [123, 124], suggesting its potential as a biomarker for early detection [125, 126] and prognosis [127]. A recent clinical case study reported that the high level of exosomal glypican-1 detected in a patient only showed progression one year after its detection [128]. Combining exosomal glypican-1 and serum CA19-9 may provide better diagnosis [129, 130]. Contrary, Lucien et al. reported they could not distinguish PDAC from benign pancreatic diseases by exosomal glypican 1 and 2 [131]. Other candidates have been reported, such as MIF [132], EphA2-EVs [133, 134] and EGFR [135, 136], epidermal growth factor receptor pathway substrate 8 (Eps8) [137], CKAP4, a DKK1 receptor [138], DCBLD2 [139] and ALPPL2 [140] which are overexpressed in circulating exosomes and can serve as biomarkers for PDAC. Exosomal c-Met and PD-L1 expression correlate with shorter postoperative survival time and serve as a negative prognostic indicator in combination with detection of CA19-9 [141]. A higher glycosylation level of CD133 in ascite-derived exosomes could indicate advanced PDAC [142]. PDAC-derived exosomal EpCAM shows an increasing expression during treatment and hence represents a useful prognostic factor [143]. As described above, tissue factor (TF) has been reported in PDAC-derived EVs and leads to venous thrombosis via interacting with endothelial cells in patients. The TF^+^ EVs are reported as biomarkers for survival of cancer patients and venous thrombosis in PDAC patients [144, 145]. Mutations on KRAS and TP53 have been well studied in PDAC and show important roles in pancreatic tumorigenesis. Both DNA and protein of mutant KRAS and TP53 are detected in circulating exosomes of patients, suggesting a potential of being biomarkers for PDAC [146–148].

**Fig. 3:**
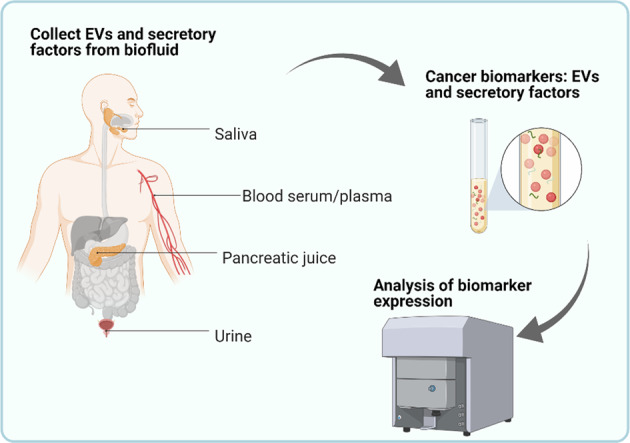
Future perspectives of PDAC early detection and diagnosis. EVs isolated from biofluid, including saliva, peripheral blood serum or plasma, pancreatic juice, and urine, provide specific exosomal protein and miRNA profiling that can distinguish PDAC patients and healthy donors. Combination of novel exosomal factors (e.g. exosomal glypican-1) and known PDAC markers, such as CA19-9 from serum, provide new strategies for earlier and more reliable PDAC diagnosis.

miRNA and long non-coding RNAs (lncRNAs) are also detected in exosomes. These nucleic acids are more resistant to RNA degrading enzymes than those present in free circulation, which allows for a higher detection sensitivity and specificity. For example, expression profiling of lncRNAs that revealed high upregulation in liver cancer (HULC) was also found highly expressed and induced by TGFβ in PDAC serum exosomes compared to healthy individuals or Intraductal papillary mucinous neoplasm (IPMN) patients [149, 150]. Numerous findings suggest novel biomarkers for early PDAC detection using different miRNA profiling from circulating exosomes (Table 2) [151–159]. The expression of serum exosomal miR-23b-3p shows correlation with serum CA19-9 level in PDAC patients [160]. Exosomal miR-10b and miR-30c signature is stated to be superior to exosomal GPC1 or plasma CA19-9 levels for PDAC diagnosis and differentiating between PDAC and CP [161]. Other exosome sources including saliva [162], pancreatic juice [163] and urine exosomes [164] are also studied to generate exosomal miRNAs profiling for PDAC detection (Fig. 3).

**Table 2:** Outline of PDAC-derived circulating exosomal miRNAs and clinical values.

Exosomal miRNAs	Expression	Cohorts	Clinical values	Ref.
miR-1246	Up	131 PDAC 25 chronic pancreatitis 22 benign pancreatic tumours 12 non-PDAC 30 volunteers	A panel of minimally invasive diagnostic biomarkers.	[151]
miR-4644	Up			
miR-3976	Up			
miR-4306	Up			
miR-18a	Up	36 PDAC 27 pre-operative PDAC 9 non-operative patients 30 control	A non-invasive cancer screening marker.	[152]
miR-196b/LCN2/TIMP1	Up	50 PDAC 20 familial pancreatic cancer 10 chronic pancreatitis 11 relevant precursor lesions 5 non-relevant lesions 20 control	A detective biomarker set for individual risk of familial pancreatic cancer.	[153]
miR-192-5p	Up	129 PDAC 107 normal control	A panel of non-invasive diagnostic markers.	[154]
miR-19a-3p	Up			
miR-19b-3p	Up			
miR-21	Up	36 PDAC 65 healthy controls	A non-invasive diagnostic biomarker and able to distinguish early-stage and advanced-stage of PDAC from healthy controls.	[155]
miR-451a	Up	56 PDAC 3 healthy controls	A potential minimally invasive biomarker for the prediction of recurrence and prognosis in PDAC.	[156]
miR-4525	Up	55 PDAC	Potential biomarkers identifying patients at high risk for recurrence and poor survival in resected PDAC patients.	[157]
miR-451a	Up	20 healthy volunteers		
miR-21	Up			
miR-191	Up	32 PDAC 29 intraductal papillary mucinous neoplasm 22 controls	A panel of early diagnostic and progression markers of PDAC.	[158]
miR-21	Up			
miR-451a	Up

## Challenges of exosome-based therapies

One long-standing strategy of cancer treatments is targeting secretory proteins and their receptors that are involved in tumorigenesis. As exosomes also play an orchestral role in tumour microenvironment, targeting exosomes provides a new approach in cancer therapies. Especially, exosomes provide protection to those cargos from antagonist treatment and enter recipient cells bypassing receptors, which can escape from current therapies and eventually cause tumour relapse. This might explain why a combination of drugs targeting both secretory proteins and their receptors increase patient survival in the first instance, but the disease still progresses later. One exosome-based strategy can be interfering exosome biogenesis and uptake. Exosome biogenesis can go through ESCRT-dependent and independent mechanisms, and inhibition of both mechanisms has shown significant suppression of cancer invasion and progression. Secondly, specific surface proteins on tumour-derived EVs which are responsible for exosome uptake regulation can be targeted. CD47, for example, is an integrin transmembrane molecule that shields exosomes from phagocytosis and facilitates micropinocytosis [165]. While exosomes have been largely described as tumorigenesis promoters, it is conceivable that exosomes also possess antitumor functions and act to restrain disease progression. Exosomes loaded with interferon-γ from dendritic cells facilitate anticancer immune responses in NK and T cells [166]. Also, constrained lncRNA SBF2-AS1 in M2 macrophage-derived exosomes inhibit PDAC progression [100]. Exosomes from NKs [101] and BM-MSCs [102–106] exert tumour suppression abilities too. This suggests an additional route in line with immune cell therapy against cancers. Intriguingly, an increasing number of studies are investigating drug-loaded exosomes as a cancer therapy. Gemcitabine and paclitaxel loaded exosomes enhanced anticancer efficacy of therapeutics [167, 168]. This suggests that exosomes can serve as vesicles since drugs incorporate during exosome biogenesis. Liposome-based drug delivery has been one of the earliest efforts. Nevertheless, the drug loading capability, the release of payload, and antitumor efficacies are varied in donor cell types [169]. Therefore, these major obstructions remain to revolutionize therapeutic advances.

## Conclusion

The evidence included in this review demonstrates that exosomes impact multiple hallmarks of PDAC progression, revealing their contribution to niche construction, metastasis, immune regulation, chemoresistance and potential for diagnosis, prognosis and therapy. However, heterogenous characteristics of exosomes make accurate exosome isolation and precise detection of exosomes still challenging. Understanding how exosomes deliver signals and how this reshapes and reprograms target cells into more malignant phenotypes provide important insight into PDAC detection and treatment.
